# Clinical outcomes and survivorship of cementless triathlon total knee arthroplasties: a systematic review

**DOI:** 10.1186/s42836-022-00124-9

**Published:** 2022-06-03

**Authors:** Brian J. Carlson, Adam S. Gerry, Jeffrey D. Hassebrock, Zachary K. Christopher, Mark J. Spangehl, Joshua S. Bingham

**Affiliations:** 1 Department of Orthopedics, Mayo Clinic Arizona, 5777 E Mayo Boulevard, Maricopa, Phoenix, AZ 85260 USA; 2 Elson S. Floyd College of Medicine, Washington State University, 412 E Spokane Falls Blvd, Spokane, Whitman, WA 99202 USA; 3 Midwestern University, Arizona College of Osteopathic Medicine, 19555 N 59th Ave. Glendale, Los Angeles, AZ 85308 USA

**Keywords:** Total knee arthroplasty, Cementless, Outcomes, Survivorship, Triathlon

## Abstract

**Background:**

Over the last decade, cementless total knee arthroplasty has demonstrated improved outcomes and survivorship due to advances in technologies of implant design, manufacturing capabilities, and biomaterials. Due to increasing interest in cementless implant design for TKA, our aim was to perform a systematic review of the literature to evaluate the clinical outcomes and revision rates of the Triathlon Total Knee system over the past decade.

**Methods:**

A systematic review of the literature was conducted following PRISMA guidelines for patients who underwent total knee arthroplasty with cementless Triathalon Total Knee System implants. Patients had a minimum of two-year follow-up and data included clinical outcome scores and survivorship data.

**Results:**

Twenty studies were included in the final analysis. The survivability of the Stryker Triathlon TKA due to all causes was 98.7%, with an aseptic survivability of 99.2%. The overall revision incidence per 1,000 person-years was 3.4. Re-revision incidence per 1,000 person-years was 2.2 for infection, and 1.3 for aseptic loosening. The average KSS for pain was 92.2 and the average KSS for function was 82.7.

**Conclusions:**

This systematic review demonstrated excellent clinical outcomes and survivorship at a mean time of 3.8 years. Additional research is necessary to examine the long-term success of the Stryker Triathlon TKA and the use of cementless TKAs in obese and younger populations.

**Level of evidence:**

III.

## Background

Total knee arthroplasty (TKA) is an effective treatment for patients with advanced arthritis of the knee and has yielded excellent results, including long-term survivorship, improved quality of life and pain relief [1–4]. Currently, there are two methods to achieve implant fixation in TKA: cemented and cementless. The cemented TKA has a long track record, and has become the gold standard but is associated with increased rates of revision and complications in both obese and young populations [5, 6]. According to registry data of 22,298 TKAs, implant revisions increased with an increasing BMI. There was a 3% increased risk of any reoperation for each unit increase in BMI, with that number jumping to 5% per unit increase when BMI was above 30 kg/m^2^ [2, 7]. A study involving 32,000 TKAs from a Finnish Arthroplasty Register showed an overall aseptic survival rate of 92% in patients younger than 55, compared to a rate of 97% in patients older than 65 [8]. It is estimated that by 2030 the projected amount of TKA performed in the United States will be approximately 3.5 million per year [9]. TKAs are also being performed in younger and more physically active patients [10]. With this in mind, interest has been mounting in cementless TKA designs that will yield positive results in these populations while maintaining a low revision rate at the same time [11, 12].

Proponents of cementless fixation argue that it has the potential for prolonged survival due to the possibility of osteointegration and long term fixation [13–15]. Although earlier studies on cementless fixation showed higher rates of aseptic loosening and revisions [16–19], recent studies have exhibited improved outcomes and survivorship due to advances in technologies of implant design, manufacturing capabilities, and biomaterials [20–26].

**Fig. 1:**
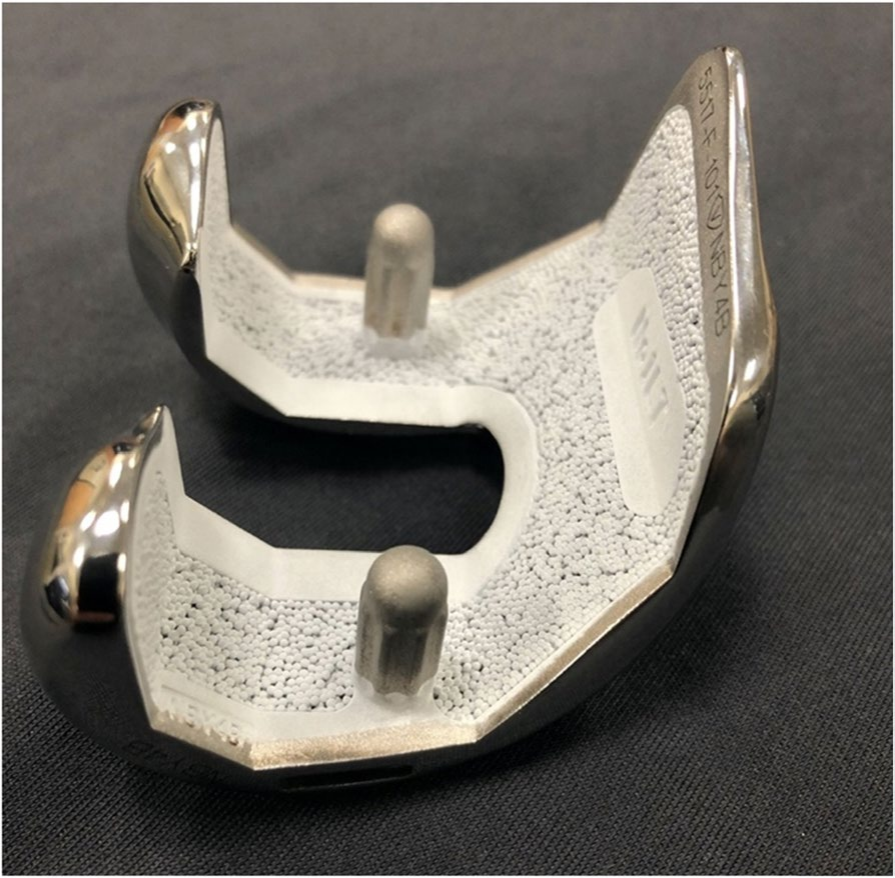
Triathalon cementless femoral component

The goal of cementless TKA is to attain biological fixation between the bone and porous implant [27]. Within the last decade, several designs have been implemented in cementless TKA to better achieve this osteointegration. Changes in various components of the implant range from the type of coating used, alterations of the pegs used to secure the baseplate to the tibia, and additive manufacturing of new porous materials. One of such variations of the cementless TKA implant is the Triathlon Total Knee System (Stryker Orthopaedics, Mahwah, New Jersey). This implant design achieves fixation by coating the cobalt-chromium beads of the femoral implant with a manufactured form of hydroxyapatite known as Peri-Apatite, which has been shown to decrease implant migration (Fig. 1) [21, 28]. Three-dimensional printing and additive manufacturing techniques are also employed to create a tibial base plate. Due to increasing interest in cementless implant design for TKA we sought to perform a systematic review of the literature to evaluate the clinical outcomes and revision rates of the Triathlon Total Knee system over the past decade. This system is one of the more popular cementless TKA designs and by focusing on one implant design variability in survivorship among different brands can be eliminated. Our objective was to perform a systematic review of the literature to evaluate the clinical outcomes and revision rates of the Triathlon Total Knee system over the past decade.

## Methods

A systematic search of PubMed and MEDLINE was conducted for English language articles published from 2005 to 2021. A combination of the terms “total knee arthroplasty”, “cementless”, “uncemented”, “non-cemented”, “clinical outcomes”, “clinical scores”, and “survivorship” were used as keywords in connection with AND or OR. Level 1 to 4 studies were included in the search. The reference list of the resulting articles was reviewed for inclusion. Each database was last searched in August 2021. Search results were exported and uploaded into a shared file and two authors (BC, AG) independently reviewed each citation and voted for it to be included or excluded from the review. If the votes were split the article would be evaluated by a third author for potential inclusion in the study.

Inclusion criteria included: (1) studies published in English, (2) age and sex of all patients reported, (3) human studies, (4) mean follow-up time longer than or equal to two years, (6) availability of clinical outcome scores, implant details and survivorship data, (7) indication of which implants components were cementless, respective outcome data, and (9) use of Triathlon Total Knee System implants.

Exclusion criteria included: (1) non-clinical studies, (2) exclusively radiostereometric analysis (RSA) studies with follow-up of less than two years, or studies that did not differentiate outcome data if multiple implant types or fixation techniques were used. Each study was evaluated in terms of methodology, patient population, and completeness of datasets. Risk of bias was addressed through assessing random sequence generation, patient selection, attrition, and selective reporting for each citation.

**Fig. 2:**
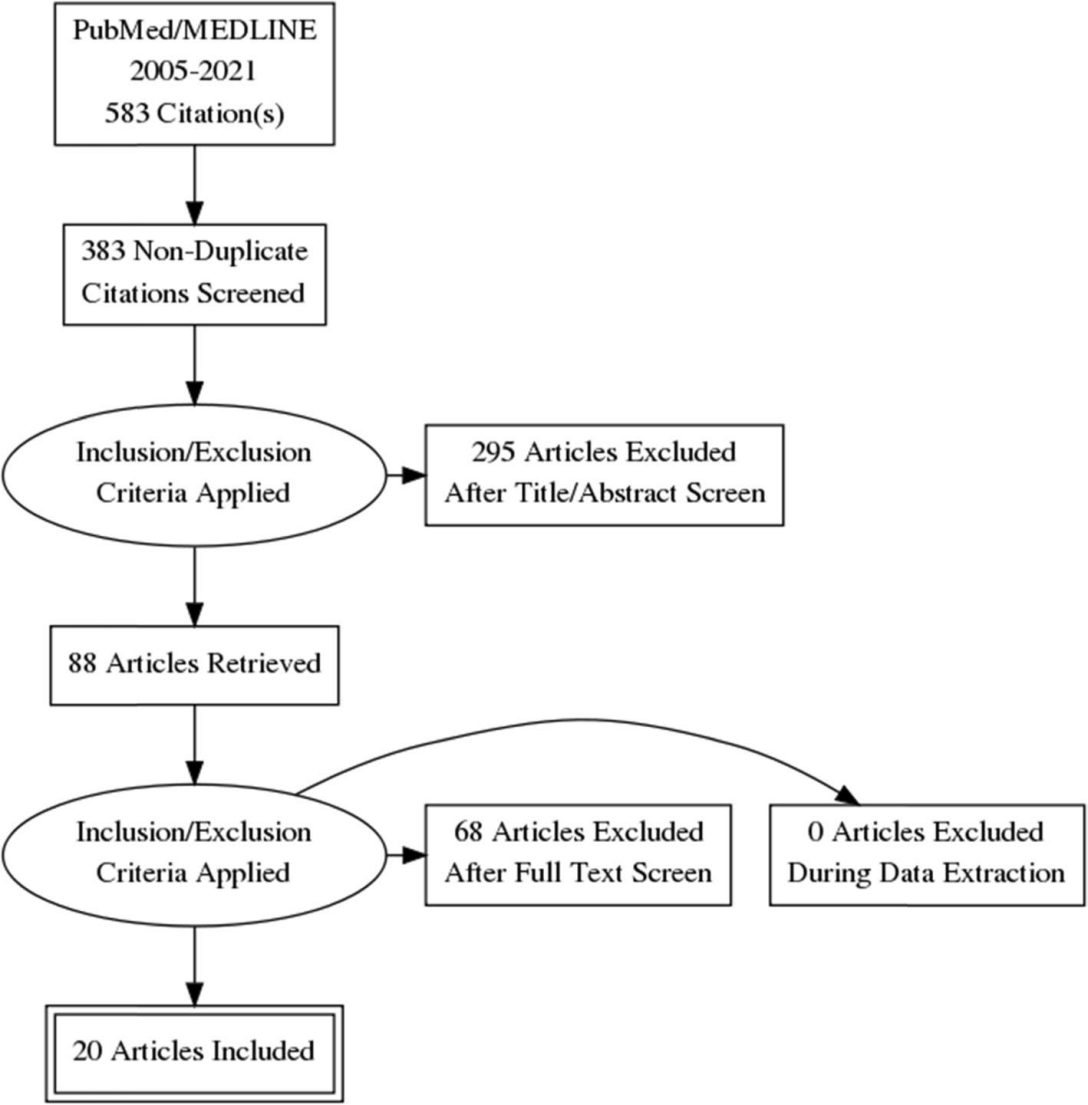
Flowchart showing the search strategy for study identification

The initial search yielded 583 citations (Fig. 2). After removal of duplicates and a review of all article titles and abstracts, a total of 88 studies were included for full-text review. After excluding studies based on follow-up time, implant brand, and inadequate outcomes, 20 studies remained for the final analysis [11, 20, 21, 29–46] (Table 1).

**Table 1:** Studies included in the systematic review for analysis

Study	Cementless TKA (*n*)	Follow-up (y)	Survivorship % at last follow-up (aseptic)	Survivorship % at last follow-up (all-cause)	Type of study
Harwin *et al* (2013) [34]	114	3	100.0%	100.0%	Prospective Consecutive Series
Harwin *et al* (2015) [36]	1025	4	99.6%	99.1%	Prospective Consecutive Series
Bagsby *et al* (2016) [11]	145	3.65	98.0%	98.0%	Multicenter Review
Harwin *et al* (2017) [39]	1024	4.4	99.5%	99.5%	Retrospective Review
Miller *et al* (2017) [41]	200	2.3	96.5%	96.5%	Retrospective Matched Case–Control
Mont *et al* (2017) [37]	31	4	100.0%	100.0%	Retrospective Review of Prospectively Collected Database
Newman *et al* (2017) [32]	142	4	99.3%	98.6%	Prospective Review
Sultan *et al* (2017) [30]	49	3.8	97.9%	96%	Prospective Review
Van Hamersveld *et al* (2017) [21]	30	5	100.0%	100.0%	5 yr Follow-Up of a Randomized Control Trial
Nam *et al* (2017) [40]	66	1.4	100.0%	100.0%	Retrospective Review of a Consecutive Series
Harwin *et al* (2017) [29]	107	8	100.0%	100.0%	Prospective Consecutive Series
Boyle *et al* (2018) [43]	154	5.9	99.4%	98.1%	Retrospective Review
Cohen *et al* (2018) [33]	72	3	100.0%	100.0%	Non-Randomized Prospective Review
Patel *et al* (2018) [38]	126	4	99.2%	99.2%	Retrospective Review
Sinicrope *et al* (2019) [35]	108	5.9	100.0%	100.0%	Retrospective Review
Nam *et al* (2019) [20]	76	2.1	99.0%	99.0%	Prospective Randomized Control Trial
Sultan *et al* (2020) [31]	568	3	98.2%	98.2%	Retrospective Review
Yazdi *et al* (2020) [42]	699	2.76	99.3%	99.3%	Retrospective Review
Hasan *et al* (2020) [45]	35	2	97.1%	97.1%	Randomized Control Trial
Restrepo *et al* (2021) [44]	341	5.5	97.1%	97.1%	Retrospective Registry Review

The data were collected after a review of the study design, the number of patients, and the risk of bias within each study. Of the included studies, 3 were randomized controlled trials [21, 40, 45], 3 were prospective non-randomized studies [34, 36, 44], 1 was a retrospective comparative study [42], 6 were retrospective case series [29–32, 37, 38], and 7 were case-control studies [11, 33, 35, 40, 41, 43]. The following data were collected from each study: the number of TKAs and implant design used for cementless fixation, including hybrid designs with individual component fixation data, mean age and follow-up of patients, sex distribution, implant survivorship, reasons for revision, complications, and clinical outcome scores. The results from the individual studies were combined for the analysis. Poisson regression analysis was used to determine the *P*-values for the incidence rates per 1,000 person-years for revisions. Cemented TKAs, Hybrid constructs and revision TKAs were excluded in the final analysis.

## Results

The 20 studies included a total of 5,112 TKAs performed on 4,873 patients (Table 2). Of the 17 studies that included preoperative diagnosis, 4,026 (78.8%) were performed because of worsening osteoarthritis, 392 (7.6%) were performed because of rheumatoid arthritis, 83 (1.6%) were performed because of knee osteonecrosis, and 611 (20%) were done for an unknown reason. The mean age at the time of surgery was 64 years (32–91) and the mean body mass index (BMI) was 33.4 kg/m^2^ (14 kg/m^2^–66 kg/m^2^). The mean follow-up time was 3.8 years (45.5 months) with the longest time being 8 years and the shortest 1.4 years. There were 1,838 male patients (37.7%).

**Table 2:** Clinical characteristics for Stryker Triathlon cementless TKA fixation

Articles	20
TKA (*n*)	5112
Patients	4837
Mean Age (y)	64
Male patients	1838 (36%)
Mean Follow-Up (*yr*)	3.8
Type of Surgery	5112 Primary (100%) 0 Revision (0%)
Reason for Surgery	4026 OA (89.4%) 392 RA (8.7%) 83 KO (1.8%) 611 unknown (12.0%)

There were a total of 68 (1.33%) revisions from all 20 studies (5,112 TKAs), with 43 (0.8%) done due to aseptic loosening and 25 (0.5%) performed due to septic failure. The survivability of the Stryker Triathlon TKA due to all causes was 98.7% with an aseptic survivability of 99.2%. The overall revision incidence per 1,000 person-years was 3.4. Re-revision incidence per 1,000 person-years was 2.2 for infection, and 1.3 for aseptic loosening. There were 61 documented complications after surgery with the most common complications being a deep vein thrombosis (18), followed by pulmonary embolism (15), postoperative stiffness MUA (9), and superficial wound necrosis (6). The overall complication rate was 1.8%.

There were a total of eight patella revisions to cemented components across the 20 studies included in this review. One patella was dislodged during manipulation under anesthesia and was replaced with a cemented component [29]. In another study, a patient had a traumatic patellar injury but was treated non-surgically and did not need revision [32]. Three studies did not resurface the patella during surgery [21, 40, 45]. Other studies used a combination of cemented and cementless patellar components and resurfacing was done at the discretion of the operating surgeon [11, 40, 42–44].

There were only two femoral component revisions across all 20 studies. One was a femoral patellar revision and the other was an all-component revision [44]. All other revisions referenced in this study were of the tibial component.

There was a broad range of data related to outcome measures in the studies included. Nineteen of the twenty studies included patient-recorded outcome measures (PROMs). Sixteen of the twenty studies collected Knee Society Scores (KSS) for pain and 15 studies collected Knee Society Scores for Function postoperatively from their patients (Table 3). The average KSS for pain was 92.2 (100) and the average KSS for function was 82.7 (100). One study [43] used the Lower Extremity Activity Scale (LEAS). Three studies collected Forgotten Joint Scores (FJS-12) [20, 43, 45], with an average score of 61.1 (100). Three studies collected Oxford Knee Scores [20, 33, 40], with an average score of 42 (48). One study [44] used the Knee Injury and Osteoarthritis Outcome for Joint Replacement (KOOS JR) score, reporting a postoperative score of 84.12 (100). Another two studies [21, 45] used KOOS and its five subscales to quantify function in addition to KSS. Two studies collected 12-item Short Form Survey (SF-12) data, with the postoperative average physical health score being 48.7 (US Average = 50). One study [35] did not collect postoperative functional outcome scores.

**Table 3:** Stryker triathlon cementless TKA functional outcomes, complications, and revision rates

Mean Knee Society Score (*n* = 14)	90.2 (100)
Mean Knee Society Functional Score (*n* = 15)	82.7 (100)
Knee Injury and Osteoarthritis Outcome for Joint Replacement (*n* = 1)	84.1 (100)
Oxford Knee Score (*n* = 3)	42 (48)
Lower Extremity Activity Scale (*n* = 1)	10.2 (18)
Forgotten Joint Score (*n* = 3)	60.1 (100)
12-item Short Form Survey (SF-12) PH (*n* = 2)	48.8 (50)
Complications (*n* = 16)	61 (1.2%), 18 DVT (0%), 15 PE (0%), 9 Stiffness (0%), 6 Superficial necrosis (0%), 4 non-displaced interop periprosthetic function (0%), 3 MI (0%), 2 Peroneal Nerve Palsy (0%), 1 Hemarthrosis (0%), 1 Deep Infection (0%), 1 Patellar tendon rupture (0%), 1 post-traumatic patellar tendon fracture (0%)
Person-Years	19,625
Revision Incidence per 1,000 person-years	3.4
Aseptic Loosening Incidence per 1,000 person-years	1.3
Septic/Infection Incidence per 1,000 person-years	2.2
Septic/Infection	25 (0.05%)
Aseptic loosening	42 (0.8%)

## Discussion

**Fig. 3:**
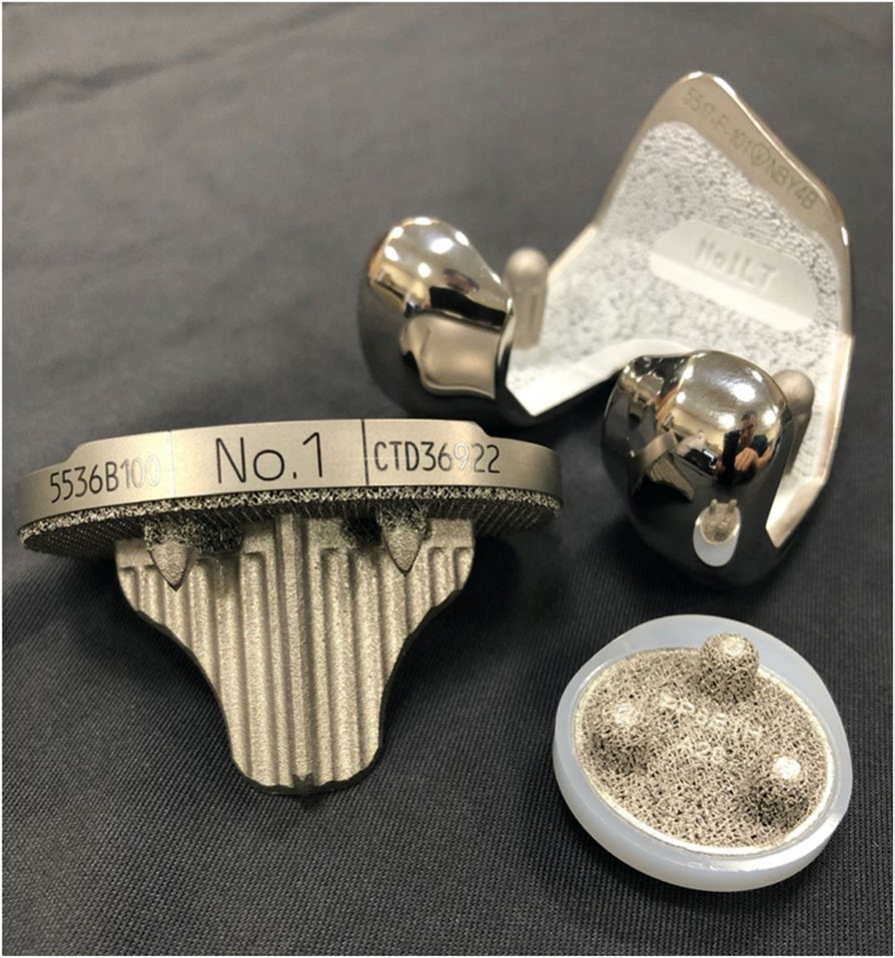
Triathlon cementless implant

**Fig. 4:**
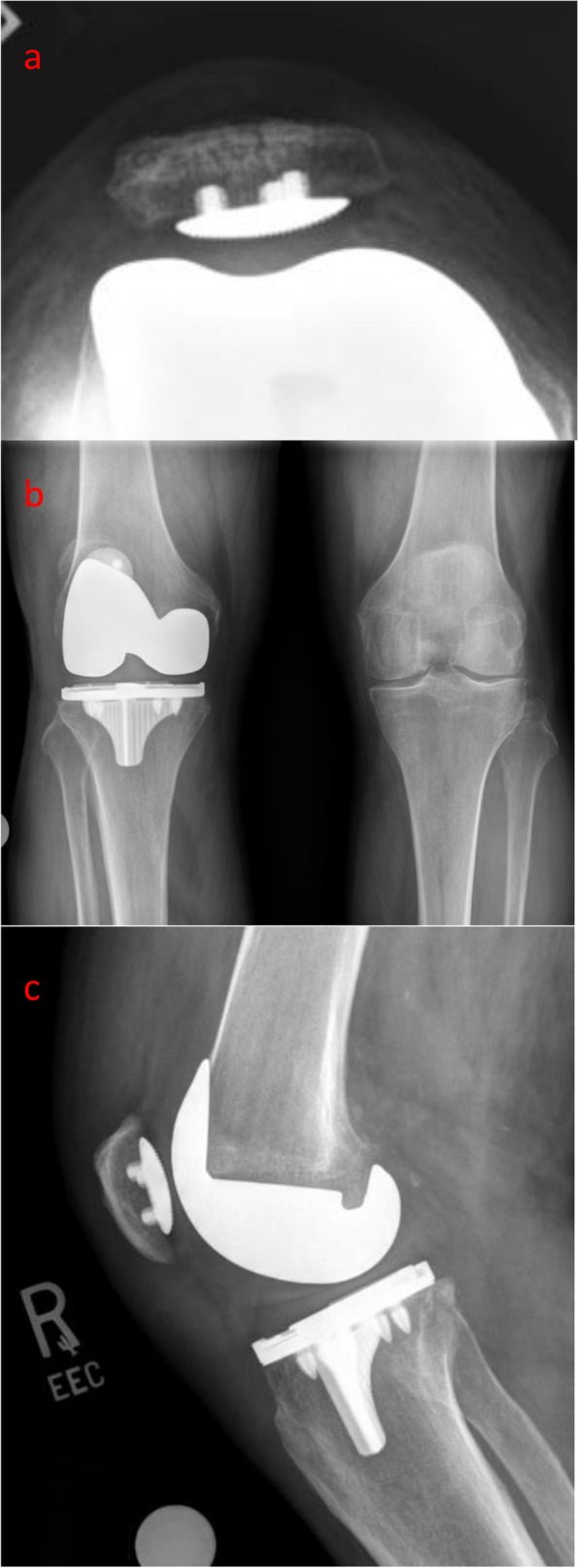
Sunrise, anteroposterior and lateral view of bone prosthetic interface

For decades cemented TKAs have been widely accepted as the gold standard for total knee replacements [47]. Recent advancements in additive manufacturing and designs of implants, which used to be too complicated to mass produce, have led to to change in opinions on the viability of cementless TKAs. This study was conducted to investigate the clinical outcomes of one specific newer-generation cementless design, the Stryker Triathlon cementless TKA implant (Fig. 3). Mounting evidence supports the newer cementless components as a viable alternative to cemented implants and may theoretically have advantages in the long run. Bagsby *et al*. [11] found better PROMs and a lower revision rate when examining the morbidly obese patient undergoing TKAs with a mean follow-up time 3.6 years. Sinicrope *et al.* [35] found that morbidly obese patients had a higher rate of operative failure due to aseptic loosening with a cemented TKA and decreasing survivorship over time. However, Boyle *et al*. [43] found that there were no differences in the outcomes between cement and cementless fixations in obese patients undergoing TKA for end-stage osteoarthritis. Patel *et al*. [38] found a 99.2% survivorship with mean follow-up in cementless TKA in patients with rheumatoid arthritis and showed no obvious contraindications for cementless TKA use in this population. Failure rates have also been shown to be higher using cemented fixation in younger (< 55 year old) patients due to loosening around the bone cement implant interface, which may be improved by utilizing a cementless implant (Fig. 4) [12, 48].

Hasan *et al*. [45] found that there was no difference in mean maximum total point motion measured via radiostereometric analysis between cemented and cementless TKA after the first three postoperative months. Although the cementless implants migrate more in the initial period after insertion, which might be attributed to settling of the implant, there were no long-term differences [21]. Multiple studies by Harwin *et al*. [29, 34, 36, 39] have shown excellent outcomes using the cementless Stryker Triathlon in an up to 9-year follow-up, with an all-cause survivorship rate of 98% [36].

In the 20 studies, involving 5,112 Stryker Triathlon TKAs were evaluated. The overall survival rate was 98.7% for the cementless tibial components at the time of final follow-up. There were only two documented failures of the femoral component and eight patellar component failures, both achieving a survival rate over 99.2%.

This study has certain limitations. Most studies included only had mid-term follow-up data, with an average follow-up time of 3.8 years. However, with the cementless design, osteointegration failure is an early complication and might occur prior to the mean follow-up. Not all studies reported the same PROMs data, and one study included no functional outcomes. Furthermore, not all studies reported complications after surgery so the number of complications reported in this review might underestimate the true complication rate. Despite these limitations, the cementless Stryker Triathlon TKA had excellent outcome scores.

The average BMI of the studies in this review was 33.4 kg/m^2^. This high BMI was due to an average BMI of around 30 kg/m^2^ in almost every study, which was slightly over the average BMI in the United States. Two studies looked into the outcomes of cementless TKA in a morbidly obese population whose average BMI was over 44 kg/m^2^ [11, 35].

## Conclusion

This study demonstrated excellent clinical outcomes of a single cementless implant at a mean time of 3.8 years. While these results are promising, this systematic review still emphasizes the need for further randomized clinical trials that examine the long-term revision rate of the Stryker Triathlon TKA and the use of cementless TKAs in obese and younger populations. Only with long-term studies can we determine the survivability and clinical outcomes of cementless Stryker Triathlon in TKA.

## Data Availability

All available data are provided. Additional data, if needed, may be made available upon request.

## Abbreviations

TKA: Total knee arthroplasty
BMI: Body mass index
RSA: Radiostereometric analysis
PROM: Patient reported outcome measure
